# The Effect of Arrestin Conformation on the Recruitment of c-Raf1, MEK1, and ERK1/2 Activation

**DOI:** 10.1371/journal.pone.0028723

**Published:** 2011-12-12

**Authors:** Sergio Coffa, Maya Breitman, Susan M. Hanson, Kari Callaway, Seunghyi Kook, Kevin N. Dalby, Vsevolod V. Gurevich

**Affiliations:** 1 Department of Pharmacology, Vanderbilt University, Nashville, Tennessee, United States of America; 2 Division of Medicinal Chemistry, The University of Texas at Austin, Austin, Texas, United States of America; Hungarian Academy of Sciences, Hungary

## Abstract

Arrestins are multifunctional signaling adaptors originally discovered as proteins that “arrest” G protein activation by G protein-coupled receptors (GPCRs). Recently GPCR complexes with arrestins have been proposed to activate G protein-independent signaling pathways. In particular, arrestin-dependent activation of extracellular signal-regulated kinase 1/2 (ERK1/2) has been demonstrated. Here we have performed *in vitro* binding assays with pure proteins to demonstrate for the first time that ERK2 directly binds free arrestin-2 and -3, as well as receptor-associated arrestins-1, -2, and -3. In addition, we showed that in COS-7 cells arrestin-2 and -3 association with β_2_-adrenergic receptor (β2AR) significantly enhanced ERK2 binding, but showed little effect on arrestin interactions with the upstream kinases c-Raf1 and MEK1. Arrestins exist in three conformational states: free, receptor-bound, and microtubule-associated. Using conformationally biased arrestin mutants we found that ERK2 preferentially binds two of these: the “constitutively inactive” arrestin-Δ7 mimicking microtubule-bound state and arrestin-3A, a mimic of the receptor-bound conformation. Both rescue arrestin-mediated ERK1/2/activation in arrestin-2/3 double knockout fibroblasts. We also found that arrestin-2-c-Raf1 interaction is enhanced by receptor binding, whereas arrestin-3-c-Raf1 interaction is not.

## Introduction

Arrestins were first discovered as proteins that bind active phosphorylated G-protein coupled receptors (GPCRs) and stop (“arrest”) G protein-mediated signaling [1] due to direct competition with G proteins for the cytoplasmic tip of the receptor [2], [3]. In the last 15 years arrestin interactions with many non-receptor partners have been described, suggesting that arrestins serve as versatile signaling regulators in the cell [4]. Crystal structures of all four vertebrate arrestins [5]–[8] revealed a very similar basal conformation: an elongated molecule consisting of two cup-like domains connected by highly conserved intra-molecular interactions. Many groups using a variety of methods invariably mapped receptor-binding elements to the concave sides of both arrestin domains [9]–[16]. Receptor binding induces a significant conformational change [10], [13], [17], [18], involving the release of the arrestin C-tail and other rearrangements (reviewed in [19]–[21]). Interestingly, microtubule binding, mediated by the same concave sides of the two domains [22], induces a distinct conformational rearrangement [22], [23]. Thus, in the cell arrestins exist in at least three distinct conformations, free, receptor-bound, and microtubule-bound [24], and many signaling proteins differentially bind arrestins in these states [25]–[27].

Specific mutants of both arrestin-2 and arrestin-3 mimicking microtubule-associated and receptor-bound conformations were constructed [22], [23], [25], [28]. Note that we use systematic names of arrestin proteins: arrestin-1 (historic names S-antigen, 48 kDa protein, visual or rod arrestin), arrestin-2 (β-arrestin or β-arrestin1), arrestin-3 (β-arrestin2 or hTHY-ARRX), and arrestin-4 (cone or X-arrestin; for unclear reasons its gene is called “*arrestin 3*” in HUGO database). Here we used wild type (WT) non-visual arrestins and their conformationally restricted mutants to determine the states that preferentially bind individual kinases of the c-Raf1-MEK1-ERK2 (ERK, extracellular signal regulated kinase; MEK1, dual specificity mitogen-activated protein kinase kinase 1, encoded by the *MAP2K1* gene in humans; c-Raf1, a.k.a. c-Raf, proto-oncogene serine/threonine-protein kinase encoded in humans by the *RAF1* gene) cascade in the presence or absence of activated β2-adrenergic receptor (β_2_AR). We found that the ERK2 binding to arrestin-2 and arrestin-3 dramatically increases when arrestins are associated with β_2_AR. Arrestin-2 interaction with c-Raf1 is enhanced by receptor binding, whereas arrestin-3-c-Raf1 interaction is not. MEK1 interaction also does not show clear preference for receptor-bound arrestin. Using pure proteins we present the first evidence that the interaction of arrestins with ERK2 is direct, and that it is differentially affected by receptor binding. These findings improve our understanding of arrestin-mediated scaffolding of MAP kinase cascades and pave the way to targeted manipulation of this branch of GPCR signaling.

## Results

### Non-visual arrestins directly bind ERK2 and facilitate its phosphorylation by MEK1

**Figure 1:**
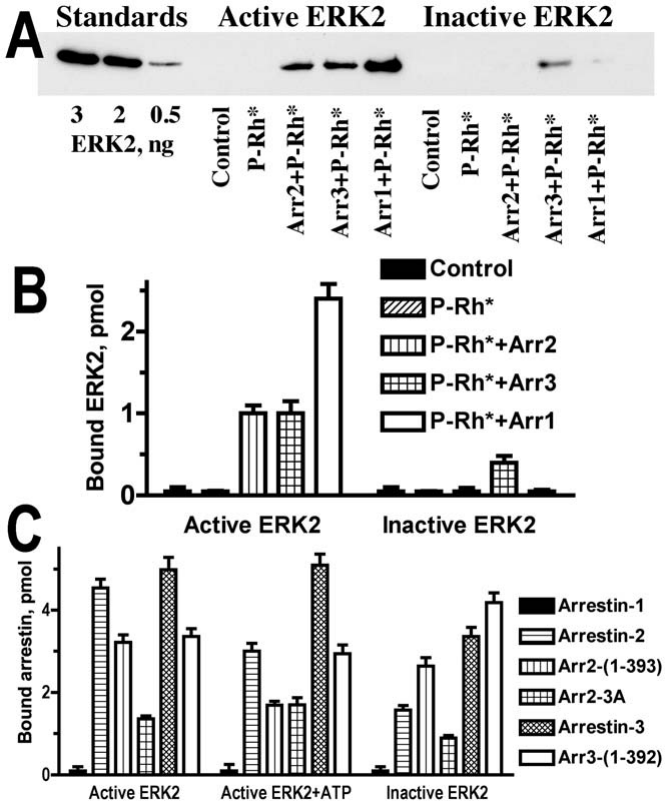
ERK2 binding to arrestin-1 and both non-visual arrestins is direct. **A**. Active (phosphorylated at T183 and Y185 by MEK1) or inactive ERK2 (30 pmol) was pre-incubated with or without 30 pmol of indicated arrestin for 20 min at 30°C, then phosphorylated rhodopsin (50 pmol) was added and incubated in the light (to produce P-Rh*) in 0.1 ml for 5 min. Rhodopsin-containing membranes were pelleted through 0.2 M sucrose cushion and dissolved in SDS sample buffer. ERK2 in the pellet (1/300 of each sample) was quantified by Western blot using anti-ERK antibodies (Cell Signaling) and known amounts of purified ERK2 to generate calibration curve. Abbreviations: Arr1, visual arrestin-1, Arr2, arrestin-2, Arr3, arrestin-3. Representative blot is shown. **B**. Quantification of ERK2 binding to P-Rh*-associated arrestins. **C**. CNBr-activated Sepharose (30 µl) containing 9 µg of covalently attached active phosphorylated (without or with 1 mM ATP) or inactive ERK2 was incubated with 3 µg of indicated purified arrestin in 60 µl of binding buffer (50 mM Tris-HCl, pH 7.4, 100 mM KCl, 1 mM EGTA, 1 mM DTT) for 20 min at 30°C. The beads were washed twice with 1 ml of ice-cold binding buffer supplemented with 0.01 mg/ml BSA. Bound arrestins were eluted with SDS sample buffer and quantified by Western blot, where known amounts of respective arrestins were run alongside samples to generate calibration curves. Means ± SD of three independent experiments are shown in panels **B** and **C**.

Although ERK2 binding to arrestins was reported a decade ago using co-immunoprecipitation [29], the proof that this interaction is direct was never presented. However, several lines of evidence suggest that ERK2 preferentially associates with receptor-bound arrestins [29]–[31]. Therefore, first we used purified proteins to test whether arrestins bound to model receptor light-activated phosphorylated rhodopsin (P-Rh*) directly interact with active (phosphorylated by MEK1) or inactive ERK2 (Fig. 1A,B). Arrestins were pre-incubated with equimolar amount of ERK2, and then allowed to bind to 1.7-fold molar excess of P-Rh* in native disc membranes. Rhodopsin-associated proteins were pelleted and the amount of ERK2 was quantified by Western blot with anti-ERK antibody. No ERK2 was detected in the pellet in the absence of rhodopsin-containing membranes or in the presence of P-Rh* alone, demonstrating that ERK2 does not appreciably bind rhodopsin. Virtually identical amount of active ERK2 phosphorylated at Thr183 and Tyr185 (PP-ERK2) was pelleted in the presence of arrestin-2 or arrestin-3 (Fig. 1A,B). Unexpectedly, even greater amount of PP-ERK2 was brought down in the presence of arrestin-1. The binding of inactive ERK2 was much lower: it was only detectable with arrestin-3. These data are the first demonstration that receptor-associated arrestins 1, 2, and 3 directly bind ERK2. All three subtypes preferentially interact with the phosphorylated form, and only arrestin-3 forms the complexes with inactive ERK2 that are stable enough to remain intact during spin-down of rhodopsin-containing membranes (Fig. 1A,B).

To test whether free arrestins also bind ERK2, we immobilized active and inactive ERK2 on CNBr-activated Sepharose, incubated beads with purified arrestins, washed, and then eluted bound proteins and quantified them by Western blot with rabbit polyclonal pan-arrestin antibody (Fig. 1C). In this format arrestin-1 was not retained by ERK2 columns, suggesting that either free arrestin-1 does not bind ERK2, or the affinity of this interaction is too low to maintain the complex throughout the washing procedure. Both non-visual arrestins bind comparably to active ERK2 (Fig. 1C). Interestingly, all “pre-activated” mutants of arrestin-2 and -3 that bind GPCRs more readily than parental wild type (WT) proteins [32]–[36] demonstrated reduced PP-ERK2 binding. The presence of 1 mM ATP in the binding assay significantly reduced the amount of full-length arrestins retained by the PP-ERK2 columns, with the exception of arrestin-3 (Fig. 1C), suggesting that inside the cell (where ∼2 mM ATP is always present) free arrestin-3 may bind ERK2 with higher affinity than arrestin-2. Interestingly, the retention of arrestin-2-3A and arrestin-3-(1–392) was not significantly affected by ATP (Fig. 1C). These mutants demonstrate greatly enhanced binding to unphosphorylated GPCRs [32], [35], [36] and even in free state appear to mimic receptor-bound conformation [37]. As was the case with receptor-associated arrestins (Fig. 1A,B), free WT arrestins show weaker binding to inactive ERK2 (Fig. 1C). Arrestin-2 appears to be significantly more selective: its binding to inactive ERK2 is ∼33% of that to active form, whereas for arrestin-3 it is ∼67%. Pre-activated mutants of both arrestins with C-terminal deletions, arrestin-2-(1–393) and arrestin-3-(1–392), are the least selective in this regard, comparably binding active and inactive ERK2 (Fig. 1C). Arrestin-3 is the most promiscuous in terms of GPCRs it binds, the least selective for active phosphorylated forms of the receptors [21], and appears to be more flexible that arrestin-2 [8]. Truncated mutants are even less selective in receptor binding [32], [33], [36], [38]. Thus, the degree of preference of different arrestins for active ERK2 correlates with their selectivity for active phospho-receptors, suggesting that increased conformational flexibility underlies the lack of selectivity in both cases.

**Figure 2:**
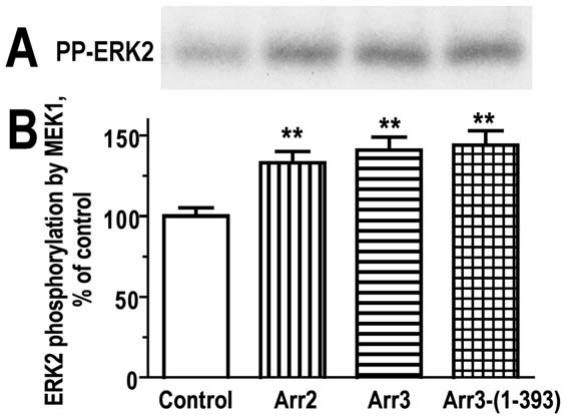
Free non-visual arrestins enhance ERK2 phosphorylation by MEK1. **A, B.** ERK2 (12 pmol) was incubated with MEK1 (2 pmol) in 0.1 ml of 50 mM Hepes-Na, pH 7.2, 100 mM NaCl, and 0.1 mM [γ-^32^P]ATP in the absence (control) or presence of 4.4 pmol of arrestin-2 (Arr2), arrestin-3 (Arr3), or arrestin-3-(1–393) (Arr3-(1–393)) for 30 min at 30°C. The reaction was stopped by MeOH-precipitation of the proteins. The pellet was dissolved in SDS sample buffer and subjected to SDS-PAGE. The gels were stained, dried, and exposed to X-ray film to visualize radiolabeled bands (panel **A**). ERK2 bands were cut out and ^32^P incorporation was quantified by scintillation counting (panel **B**). Means ± SD of four independent experiments are shown. (**) p<0.01, as compared to control.

Next, we tested whether arrestin binding affects ERK2 phosphorylation by MEK1. Purified inactive (unphosphorylated) ERK2 and purified constitutively active MEK1 (which phosphorylates ERK2) were used to reconstruct this module of c-Raf1-MEK1-ERK1/2 cascade *in vitro* (Fig. 2). ERK2 phosphorylation by MEK1 was evaluated in the absence or presence of purified arrestins. We found that in the absence of arrestins MEK1 transfers ∼2.4 pmol of phosphates. Taking into account that MEK1 phosphorylates two sites in each ERK2 molecule, this is equivalent to the phosphorylation of ∼10% of ERK2 present (Fig. 2). In the presence of arrestin-2 or arrestin-3 the extent of ERK2 phosphorylation was increased by 33 or 41%, respectively. Thus, free non-visual arrestins moderately facilitate the phosphorylation of ERK2 by MEK1. These data suggest that non-visual arrestins also bind MEK1.

### The effect of arrestin-2 and arrestin-3 binding to the β2-adrenergic receptor on its interactions with c-Raf1, MEK1, and ERK2 in cellular environment

The first report on the role of arrestins in the activation of c-Raf1-MEK1-ERK1/2 cascade suggested that only receptor-bound arrestins interact with c-Raf1 and ERK1/2, whereas MEK1 does not bind arrestins directly, but is recruited via c-Raf1 and ERK to the complex [29]. Subsequent studies showed that all three kinases bind free non-visual arrestins and even separately expressed N- and C-domains of arrestin-2 and –3 that do not bind GPCRs, and that ERK demonstrates the lowest affinity of the three [31]. MEK1 interaction with free arrestin-2 was independently confirmed by another group [39]. However, the effects of arrestin-2 and -3 conformation and receptor binding on their interaction with these kinases were never systematically investigated. Therefore, we used two known conformationally biased forms of arrestin-2 and -3, “pre-activated” 3A mutants [32], [35], [36] and mutants “frozen” in the basal state by a 7-residue deletion in the inter-domain hinge (Δ7) [22], [25], [26], [28] to address this question in COS-7 cells expressing only endogenous β2AR, or additional plasmid-encoded β2AR at significantly greater level.

**Figure 3:**
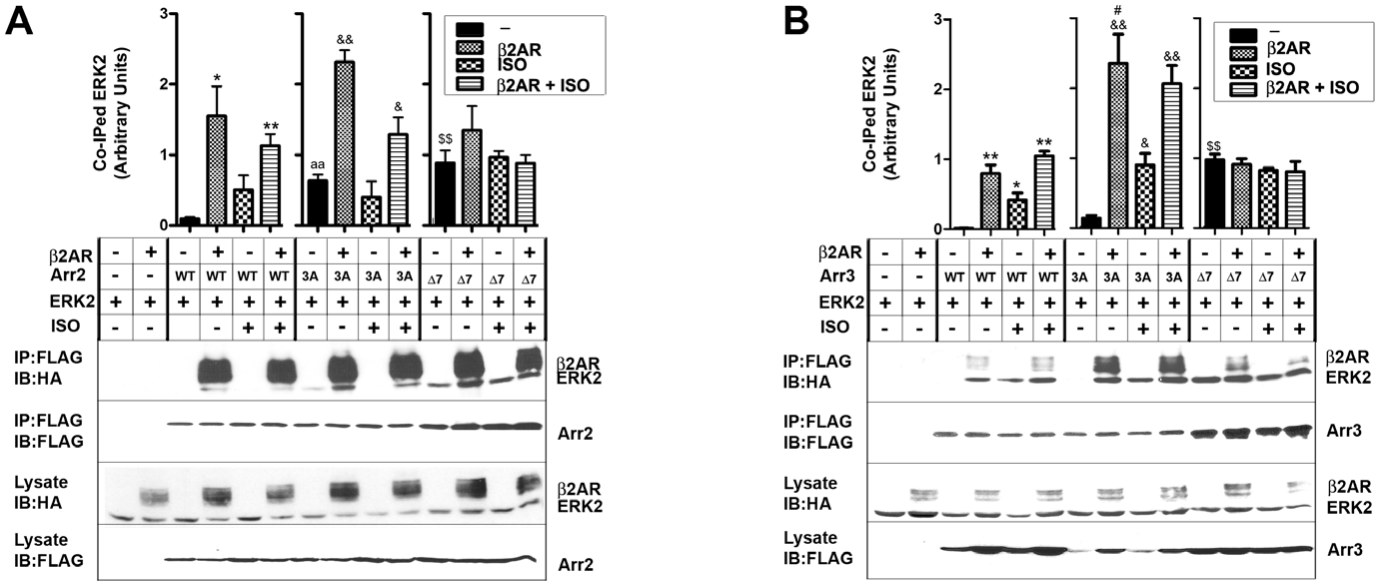
Conformational dependence of the interaction of non-visual arrestins with ERK2. COS-7 cells were transfected with WT, 3A, or Δ7 mutant forms of Flag-tagged arrestin-2 (**A**) or arestin-3 (**B**), along with ERK2-HA, with or without HA-β2AR. Cells were serum starved overnight 24 hours post-transfection and treated for 10 min at 37°C with or without 10 µM β2AR agonist isoproterenol. Cells were lysed, and arrestins were immunoprecipitated with anti-Flag antibody, and co-immunoprecipitated ERK2 and β2AR were detected with anti-HA antibody. Bar graphs show the ratio of co-immunoprecipitated ERK2 to immunoprecipitated arrestin. The data from three independent experiments were statistically analyzed by ANOVA. The significance of the differences is indicated, as follows: * or **^&^**, p<0.05; ** or **^&&^**, p<0.01, as compared to corresponding within group basal level of ERK2 co-immunoprecipitation (black bars); **^a^** or **^$^** or **^#^**, p<0.05 compared to WT control (black bar in WT group).

We found that the stimulation of endogenous β2AR by an agonist isoproterenol dramatically increased ERK2 binding to arrestin-2 and arrestin-3 (Fig. 3). Over-expression of β2AR resulted in the formation of an arrestin-receptor complex independent of isoproterenol stimulation and further increased the binding of ERK2 to arrestins (Fig. 3). Apparently, at high levels of β2AR, which is known to have significant constitutive activity [40], basal arrestin-β2AR association is fairly high and is not significantly enhanced by isoproterenol stimulation. Pre-activated 3A mutants bind ERK2 much better than corresponding wild type (WT) arrestins. Since 3A mutation forcibly detaches the arrestin C-tail [37], similar to receptor binding [10], [13], [18], which makes 3A mutants mimics of the receptor-bound state, these results are in agreement with the evidence that ERK2 preferentially binds receptor-associated arrestins (Fig. 1) [29]. Co-expression of β2AR with 3A mutants further enhanced arrestin-ERK2 interaction (Fig. 3). Unexpectedly, we found that Δ7 mutants of both arrestins also bind ERK2 significantly better than WT proteins or even 3A mutants (Fig. 3). This is consistent with reported ability of Δ7 mutants of arrestin-2 and -3 to recruit ERK1/2 to microtubules, which they bind with high affinity [22]. In agreement with impaired ability of Δ7 mutants to bind GPCRs [22], [28], we found that neither isoproterenol stimulation nor β2AR over-expression affected ERK2 binding to Δ7 forms of either arrestin (Fig. 3). Thus, ERK2 preferentially interacts with arrestins in receptor-bound and microtubule-associated conformation, whereas free arrestins in the basal state show the lowest level of association with this kinase.

**Figure 4:**
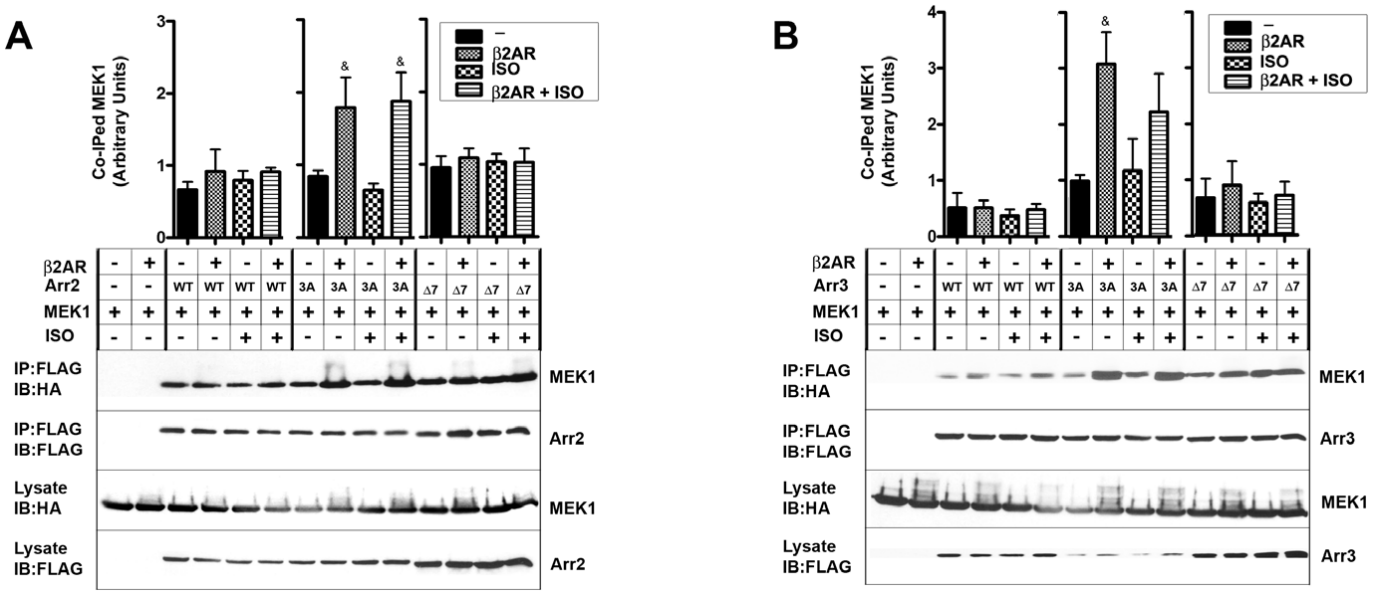
The binding MEK1 is insensitive to arrestin conformation. COS-7 cells were transfected with WT, 3A, or Δ7 mutant forms of Flag-tagged arrestin-2 (**A**) or arrestin-3 (**B**), along with MEK1-HA, with or without HA-β2AR. Cells were serum starved overnight 24 hours post-transfection and treated for 10 min at 37°C with or without 10 µM β2AR agonist isoproterenol. Cells were lysed, and arrestins were immunoprecipitated with anti-Flag antibody, and co-immunoprecipitated MEK1 and β2AR were detected with anti-HA antibody. Bar graphs show the ratio of co-immunoprecipitated MEK1 to immunoprecipitated arrestin. The data from three independent experiments were analyzed by ANOVA. **^&^**, p<0.05, as compared to corresponding within group basal level of MEK1 co-immunoprecipitation (black bars).

In contrast to ERK2 (Fig. 3), MEK1 association with both arrestins in unstimulated cells was readily detectable (Fig. 4). Isoproterenol stimulation with or without β2AR over-expression did not appreciably affect MEK1 binding to WT arrestin-2, arrestin-3, and their Δ7 mutants (Fig. 4). Interestingly, MEK1 co-immunoprecipitated with 3A mutants was dramatically increased by β2AR over-expression regardless of isoproterenol stimulation (Fig. 4). Thus, receptor binding does not significantly affect MEK1 interactions with WT arrestins, but enhances MEK1 binding to conformationally loose [37] 3A mutants. As far as interactions with ERK2 and MEK1 are concerned, no subtype-specific differences between arrestin-2 and –3 and their respective mutants were apparent in the environment of living cells (Figs. 3, 4).

**Figure 5:**
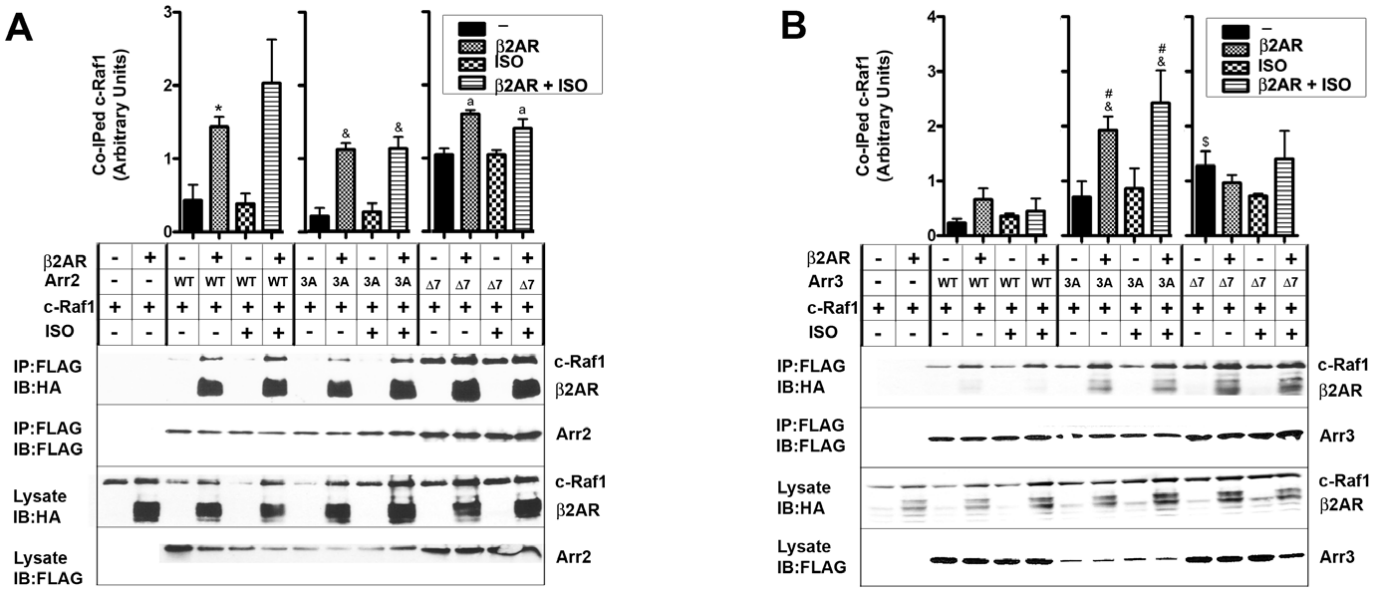
Conformational dependence of arrestin interactions with c-Raf1. COS-7 cells were transfected with WT, 3A, or Δ7 mutant forms of Flag-tagged arrestin-2 (**A**) or arestin-3 (**B**), along with c-Raf1-HA, with or without HA-β2AR. Cells were serum starved overnight 24 hours post-transfection and treated for 10 min at 37°C with or without 10 µM β2AR agonist isoproterenol. Cells were lysed, and arrestins were immunoprecipitated with anti-Flag antibody, and co-immunoprecipitated c-Raf1 and β2AR were detected with anti-HA antibody. Bar graphs show the ratio of co-immunoprecipitated c-Raf1 to immunoprecipitated arrestin. The data from three independent experiments were analyzed by ANOVA. * or **^&^** or **^a^**, p<0.05, as compared to corresponding within group basal level of c-Raf1 co-immunoprecipitation (black bars); **^$^** or **^#^**, p<0.05, compared to WT control (black bar in WT group).

In contrast to ERK2 and MEK1, the binding of c-Raf1 to WT arrestin-2 and -3 was differentially affected by β2AR over-expression (Fig. 5). The presence of extra β2AR resulted in a dramatic increase in c-Raf1 binding to arrestin-2, whereas in case of arrestin-3 receptor effect was only marginal (Fig. 5). This difference is in agreement with recent discovery that alanine substitution of R307 in arrestin-2 greatly reduces c-Raf1 binding and its ability to facilitate ERK1/2 activation in cells, whereas homologous K308A mutation in arrestin-3 does not [30]. When the two subtypes were rendered conformationally flexible by 3A mutation, β2AR over-expression comparably increased c-Raf1 binding to both non-visual arrestins (Fig. 5). Similar to ERK2 and MEK1, more c-Raf1 co-immunoprecipitated with Δ7 mutants than with WT forms of either arrestin. C-Raf1 binding to arrestin-2-Δ7 was moderately increased by β2AR over-expression, likely reflecting remaining ability of arrestin-2-Δ7 to bind receptors [22].

To summarize, isoproterenol activation of the endogenous receptor present at relatively low levels resulted in detectable increase only of ERK2 interaction with WT arrestins (Fig. 3), which was previously found to have the lowest propensity to associate with free arrestins [31]. In contrast, significant over-expression of β2AR increased the binding of ERK2 and c-Raf1, but not MEK1, to WT arrestins (Figs. 3, 4, and 5). As could be expected, in the case of 3A mutants that bind GPCRs more readily than WT proteins [32], [36], [38], [41], the interaction with all three kinases is increased by receptor over-expression, whereas Δ7 mutants impaired in receptor binding ability are essentially unresponsive to β2AR (Figs. 3, 4, and 5). Unexpectedly, we found that Δ7 mutants of arrestin-2 and -3 bind ERK2 and c-Raf1 better than parental WT arrestins (Figs. 3, 5). The same tendency was observed with MEK1, although it did not reach statistical significance (Fig. 4). Thus, the interactions of non-visual arrestins with the kinases c-Raf1 and ERK2 are very sensitive to the arrestin conformation, whereas the binding of MEK1 is minimally affected by the functional state of arrestins.

### Receptor-stimulated arrestin-dependent ERK activation

**Figure 6:**
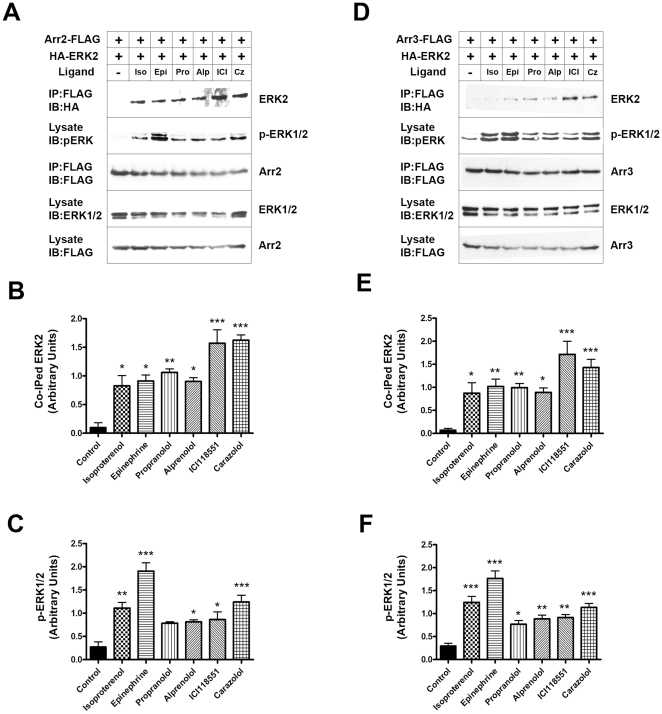
The effect of different β2AR ligands on ERK2 binding to arrestins and ERK2 activation. HA-tagged ERK2 was co-expressed with Flag-tagged WT arrestin-2 (**A,B,C**), or arrestin-3 (**D,E,F**) in COS-7 cells. Cells were serum starved 24 hours after transfection and stimulated for 10 min at 37°C with 10 µM of indicated β2AR ligands. Arrestins were immunoprecipitated with anti-Flag antibody, and co-immunoprecipitated ERK2 was visualized with anti-HA antibody. The binding of ERK2 to arrestin-2 (**B**) or arrestin-3 (**E**) was significantly increased by treatment with ligands. **C,D.** ERK1/2 activation in cell lysates was determined by Western blot with anti phospho-ERK1/2 antibody. Means ± SD of 3–4 independent experiments are shown in bar graphs; representative blots are shown in panels **A** and **D**. ANOVA with Bonferroni post-hoc test revealed the following differences: *, p<0.05; **, p<0.01; ***, p<0.001, as compared to untreated cells.

Next, we tested whether arrestin-ERK2 interaction correlates with receptor-dependent ERK2 activation. To this end, we expressed HA-ERK2 with arrestin-2-Flag (Fig. 6A) or arrestin-3-Flag (Fig. 6D) in COS-7 cells and stimulated endogenous β2AR with saturating concentrations of agonists (isoproterenol, epinephrine), antagonists (propranolol, alprenolol), or inverse agonists (ICI118551, carazolol). Unexpectedly, we found that the amount of ERK2 co-immunoprecipitated with arrestin-2 (Fig. 6B) or arrestin-3 (Fig. 6E) was significantly increased by agonists, antagonists, and inverse agonists. Since these ligands were shown to induce distinct conformational changes in β2AR [42], these data indicate that arrestins bind more than one conformational state of the receptor, and this binding promotes similar increases in ERK2 interaction. Importantly, the level of ERK2 phosphorylation was also increased by different ligands in cells expressing arrestin-2 (Fig. 6C) and arrestin-3 (Fig. 6F). Inverse agonists ICI118551 and carazolol induced the most dramatic increase in ERK2 association with arrestins and significant increase in ERK2 activation (Fig. 6), supporting the idea that these compounds are in fact arrestin-biased agonists [43]. Presumed antagonists propranolol and alprenolol (that actually have partial agonist activity [40]) also promoted ERK2 binding to arrestins and ERK2 phosphorylation, albeit to a lesser degree (Fig. 6). Agonists isoproterenol and epinephrine produced disproportionally larger ERK2 activation relative to its association with arrestins (Fig. 6), likely because, in contrast to other compounds tested, these ligands increase G protein activation, and ERK can be also activated by GPCRs via G-protein mediated pathways [44].

**Figure 7:**
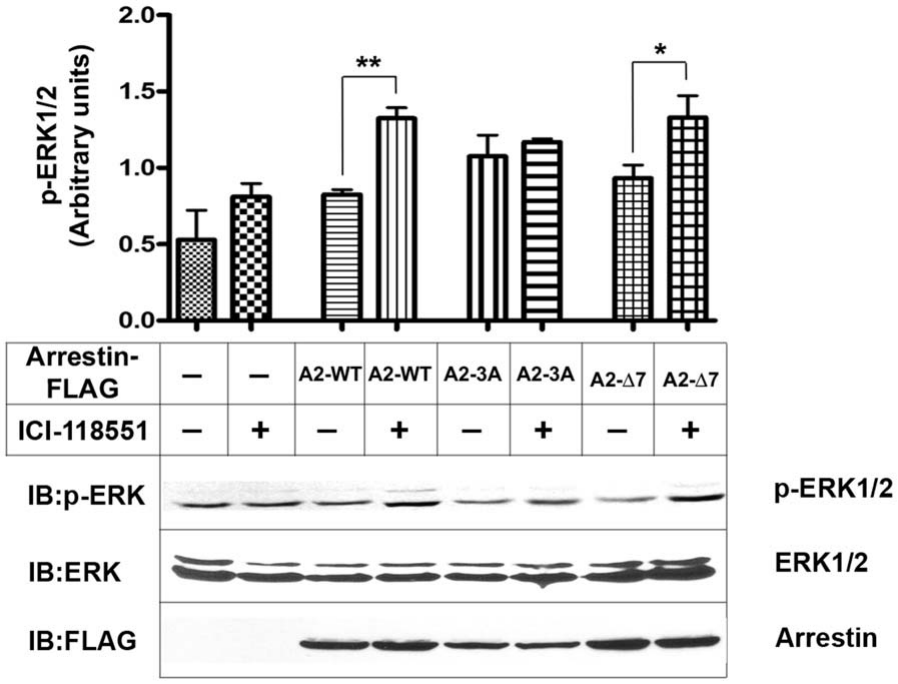
WT and Δ7 mutant of arrestin-2 rescue β2AR-mediated ERK activation in response to ICI118551 in DKO MEFs. DKO MEFs were infected with retrovirus encoding GFP (control, -), or untagged WT arrestin-2 (A2-WT), arrestin-2-3A (A2-3A), or arrestin-2-Δ7 (A2-Δ7). The cells were serum-starved 48 hours post-infection for 2 hours, stimulated with 1 µM ICI118551 for 10 min at 37°C, lysed, and analyzed by Western blot. Means ± SD of 3–4 independent experiments are shown in bar graphs; representative blots are shown below. *, p<0.05; **, p<0.01.

Therefore, to exclude G protein-mediated mechanisms, we performed the next set of experiments in arrestin-2/3 double knockout (DKO) MEFs [45], where ERK2 activation by β2AR inverse agonists is strictly arrestin-dependent. An inverse β2AR agonist ICI118551, was previously shown to act as an arrestin-biased agonist [46]. Indeed, we did not detect appreciable ERK1/2 activation by ICI118551 via endogenous β2AR in DKO MEFs (Fig. 7). We found that the expression of WT arrestin-2 rescues the ability of ICI118551 to stimulate ERK1/2 phosphorylation. Interestingly, arrestin-2-Δ7 was also effective, in contrast to arrestin-2-3A mutant (Fig. 7).

**Figure 8:**
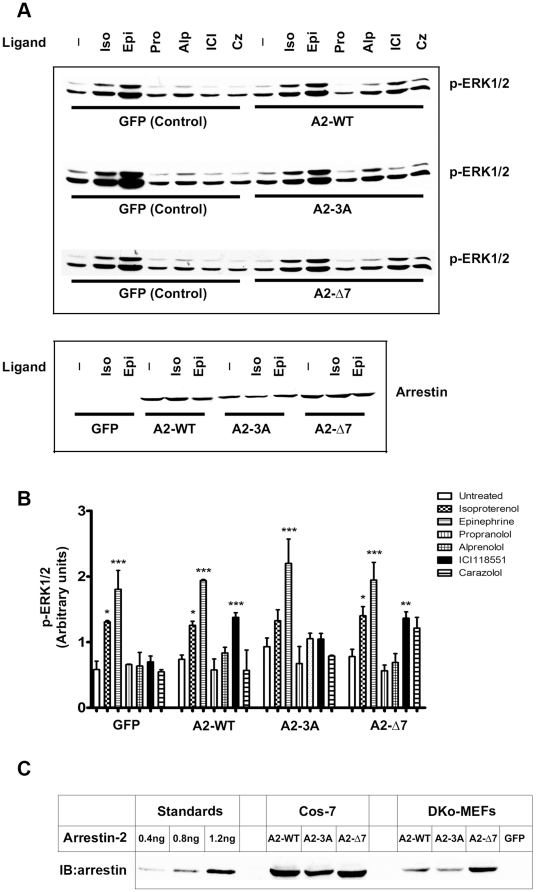
ERK2 activation by different β2AR ligands in DKO MEFs. **A.** DKO MEFs were infected with retrovirus encoding GFP, untagged WT arrestin-2 (A2-WT), arrestin-2-3A (A2-3A), or arrestin-2-Δ7 (A2-Δ7). Serum-starved cells were stimulated with indicated β2AR ligands, lysed, and analyzed by Western blot. Representative blots are shown. The expression of different forms of arrestin-2 is compared in the blot below. **B.** Phospho-ERK1/2 bands were quantified. Means ± SD of 3 independent experiments are shown. **C.** Comparison of arrestin expression levels in COS-7 cells (5 µg protein/lane) and DKO MEFs (10 µg protein/lane) was performed by Western blot with anti-arrestin antibody. Standards containing indicated amounts of purified arrestin-2 were run along with cell lysates to generate calibration curve. Arrestin expression was measured by quantitative Western in COS-7 cells: A2-WT, 100.1 pmol/mg; A2-3A, 81.1 pmol/mg; A2-Δ7, 92.8 pmol/mg. Arrestin expression in DKO MEFs was much lower: A2-WT, 13.2 pmol/mg; A2-3A, 12.3 pmol/mg; A2-Δ7, 21.7 pmol/mg.

To determine which β2AR ligands enhance ERK1/2 phosphorylation in arrestin-dependent fashion, we compared ERK1/2 activation in DKO MEFs expressing GFP (control), WT arrestin-2, as well as Δ7 or 3A mutants (Fig. 8A,B). In all cases we detected robust ERK1/2 activation in response to isoproterenol and epinephrine, further confirming that this effect is mediated by G protein, rather than arrestins. In this model the antagonists propranolol and alprenolol did not affect ERK1/2 phosphorylation regardless of arrestin expression (Fig. 8). Only cells expressing WT arrestin-2 and Δ7 mutant showed ERK1/2 activation in response to ICI118551; however, we did not detect a statistically significant response to carazolol (Fig. 8A,B), which activated ERK1/2 in COS-7 cells over-expressing arrestins (Fig. 6). To determine possible reason for this difference, we compared the expression of arrestins in COS7 cells and DKO-MEFs, and found that the latter express all arrestins at much lower levels (Fig. 8C). Thus, ICI118551 appears to be more potent activator of arrestin-mediated signaling, effective even at fairly low arrestin expression levels.

## Discussion

In addition to classical G protein-mediated signaling, GPCRs were shown to initiate several signaling pathways via bound arrestins, which lead to the activation of ERK1/2 [29], c-Jun N-terminal kinase 3 (JNK3) [47], and p38 [48]. The ERK1/2 activating module consists of three kinases: c-Raf1 phosphorylates MEK1, which in its turn phosphorylates ERK1/2 on both tyrosine and threonine residues [49] within the activation loop. ERK1/2 activation by GPCRs can be mediated by the activation of Ras, PKC, tyrosine kinases (e.g., c-Src), trans-activation of receptor tyrosine kinases, or via arrestins. ERK1/2 activity controls many cellular functions, including proliferation, differentiation, and apoptosis. Arrestin-mediated ERK1/2 activation may result in different physiological responses than those achieved by G protein activation. G protein activation of ERK1/2 results in the translocation of active ERK to the nucleus, where it can phosphorylate and activate various transcription factors [50]. In contrast, when ERK1/2 is activated via arrestin-dependent mechanism, active ERK1/2 largely remains in the cytoplasm, where it can phosphorylate non-nuclear substrates [51].

Although non-visual arrestins were proposed to act as scaffolds for the c-Raf1-MEK1-ERK1/2 cascade, their direct interactions with any of these kinases were never experimentally demonstrated. Here using purified proteins we have unambiguously shown for the first time that ERK2 directly binds arrestin-1, -2, and -3 (Fig. 1). The experiments with pure proteins under strictly controlled conditions revealed that all receptor-bound arrestins recruit active (phosphorylated by MEK1) ERK2 more efficiently than inactive ERK2, and that arrestin-1 is the most selective, whereas arrestin-3 is the least selective in this regard (Fig. 1). These results are compatible with the model where receptor-associated arrestin facilitates ERK1/2 phosphorylation and retains generated active ERK in the complex, which would localize active ERK to the cytoplasm [29], [52], in contrast to ERK activated via other mechanisms, which translocates to the nucleus. This is the first plausible mechanistic explanation for cytoplasmic localization of ERK1/2 activated via arrestin-dependent mechanism. We also detected measurable interaction of active and inactive ERK2 with free arrestin-2 and –3, but not with free arrestin-1 (Fig. 1C), which suggests that non-visual arrestins can come to the receptor “pre-loaded” with bound ERK, which would facilitate its activation in response to GPCR stimulation. Using purified proteins we also demonstrated for the first time that arrestins facilitate ERK2 activation by MEK1 (Fig. 2), a function that was proposed [29], [52] but never proven. The magnitude of this effect in the *in vitro* assay was modest, likely due to fairly high concentrations of ERK2, MEK1, and arrestins used. However, these proof-of-principle experiments suggest that arrestin impact is likely much greater in cells, where the “concentrating” effect of binding of the two kinases would be much stronger due to significantly lower absolute concentrations of the proteins involved. Our data are compatible with two distinct roles of arrestin. One possibility is that ERK2 binding to arrestin changes its conformation, making it a better substrate for MEK1. For example, it was recently shown using purified proteins that “scaffold” Ste5 in yeast acts by making MAPK Fus3 (but not related kinase Kss1) a better substrate for MAPKK Ste7, rather than by bringing Ste7 and Fus3 together [53]. In the second model arrestin can act as a true scaffold, bringing both MEK1 and ERK2 into close proximity to each other, thereby facilitating the phosphorylation of ERK2 by MEK1. Simple scaffolding mechanism was recently demonstrated for the arrestin-MKK4-JNK3 signaling module reconstituted from pure proteins [54]. Detailed kinetic studies of the activity of the arrestin- MEK1-ERK2 complex reconstructed from pure proteins are necessary to elucidate the exact mechanism of arrestin action.

Previously we found that ERK2 co-immunoprecipitation from cells with free arrestins is barely detectable without cross-linking [55], whereas receptor-associated arrestins readily co-immunoprecipitate with ERK2 (Fig. 3). These data suggest that ERK2 binding is highly sensitive to arrestin conformation. To gain further insight into confromational preference of ERK2, we co-expressed it with three distinct forms of arrestins: a) WT with normal conformational flexibility; b) “pre-activated” 3A mutants with detached C-tail that partially mimic receptor-bound state [32], [36]; c) Δ7 mutants with the deletion of seven residues in the inter-domain hinge, which significantly impedes receptor binding [22], [25], [28] by “freezing” arrestin in the basal conformation. Our data show that conformational change induced by arrestin recruitment to β2AR dramatically increases ERK2 binding to both non-visual arrestins (Fig. 3). Unexpectedly, we found that ERK2 also avidly binds Δ7 mutants, so that free WT arrestins appear to be its least favorite partners (Fig. 3). ERK2 binding to both non-visual arrestins shows the same conformational dependence (Fig. 3). MEK1 demonstrates much higher binding to both arrestins in their basal state and does not show appreciable conformational dependence in its interactions with arrestin-2 or -3 (Fig. 4). Indeed, in the experiments where we immunoprecipitated Flag-tagged arrestins and immunoblotted for kinases, all of which have the same HA tag as β2AR, we observed the presence of considerable amounts of co-immunoprecipitated receptor with ERK2 (Fig. 3) and c-Raf1 (Fig. 5), but not with MEK1 (Fig. 4), indicating that a significant fraction of ERK2- and c-Raf1-associated arrestin is bound to the receptor, whereas most of MEK1-associated arrestin is free. These data suggest that arrestins recruited to active phosphorylated receptors are more likely to be pre-loaded with MEK1 than with ERK. We found that arrestin-2 binding to c-Raf1 is much more sensitive to the receptor interaction than that of arrestin-3 (Fig. 5). Since distinct structural features of arrestin-3 also result in lower selectivity for particular functional forms of the receptor than that of arrestin-2 [8], these data suggest that higher conformational flexibility of arrestin-3 is responsible for more promiscuous interactions with GPCRs and other signaling proteins. Markedly different effects of receptor binding on arrestin-2 and -3 interaction with c-Raf1 are consistent with distinct ability of these subtypes to scaffold c-Raf1-MEK1-ERK1/2 cascade [56].

To determine how receptor-dependent changes in arrestin interactions with these kinases translate into agonist-dependent ERK1/2 activation, we used β2AR that is endogenously expressed in most cultured cells at physiologically relevant levels, and took advantage of the availability of arrestin-biased agonists for this receptor [43]. Relatively low levels of endogenous arrestins in COS-7 cells ensure that exogenously expressed arrestin is the predominant species. We found that the expression of WT arrestin-2 or -3, which are the most sensitive to receptor interaction (Figs. 3, 4, and 5), enhanced the phosphorylation of endogenous ERK1/2 in response to β2AR stimulation by unbiased agonists adrenaline and isoproterenol, antagonists alprenolol and propranolol that show low agonist activity [40], as well as arrestin-biased agonists carazolol and ICI118551 (Fig. 6). ERK1/2 phosphorylation induced by carazolol and ICI118551, which are inverse agonists for G protein activation, is comparable to that induced by unbiased agonists isoproterenol and adrenaline that can promote ERK activation via G proteins and arrestins (Fig. 6), suggesting that a significant fraction of ERK1/2 is activated via arrestin-mediated mechanism.

To conclusively dissect arrestin-dependent and arrestin-independent mechanisms, we compared WT MEFs, where ERK1/2 can be activated via both pathways, and DKO MEFs lacking non-visual arrestins [45], where only G protein-mediated pathway is operative. Indeed, we found that while ERK1/2 phosphorylation in response to β2AR agonists that promote receptor coupling to G protein is essentially the same, the response to ICI118551 is completely lost in DKO MEFs, indicating that it is mediated by non-visual arrestins absent in these cells (Fig. 7). The advantage of DKO MEFs is that one can be confident that the expressed form of arrestin is the only one present. For subsequent experiments we chose arrestin-2, which showed more pronounced changes in kinase interactions in response to receptor binding (Figs. 3, 4, and 5). We found that WT arrestin-2 and Δ7 mutant rescue ERK1/2 response to ICI118551 in DKO MEFs, whereas the 3A mutant does not (Fig. 7). Next we tested a wider range of β2AR ligands in DKO MEFs expressing GFP (control), WT arrestin-2, 3A, or Δ7 mutant (Fig. 8). We found that arrestin expression in DKO MEFs was 5–6 times lower than in COS-7 cells (Fig. 8C). In these conditions only ICI118551 induced robust ERK1/2 activation, indicating that it is more potent stimulator of arrestin-mediated signaling than carazolol.

To summarize, here we demonstrated for the first time that arrestins directly binds ERK2, determined the conformations of arrestin-2 and -3 preferred by c-Raf1 and ERK2, and showed that MEK1 similarly interacts with arrestins in all conformational states. We found that ERK2 and c-Raf1 interact with the arrestin-receptor complex better than with free arrestins. Unexpectedly, we also found that Δ7 mutants with significantly reduced ability to bind receptors readily interact with ERK2 and c-Raf1. Interestingly, WT arrestin-2 and Δ7 mutant comparably rescue arrestin-dependent activation of ERK1/2 in response to receptor stimulation by arrestin-biased ligands. Since dramatically reduced binding of Δ7 forms of arrestin-1, -2, and -3 was described using light-activated phosphorhodopsin [22], [28], which appears to bind G protein and arrestin equally well, our data suggest that arrestin-2-Δ7 is less impaired in binding receptors in a distinct conformation induced by arrestin-biased agonists. Further structural dissection of receptor conformations that preferentially engage G proteins and arrestins requires the solution of crystal structures of receptors in complex with these two types of partners. So far, only one structure of a GPCR with bound signaling protein, β2AR-Gs complex, has been solved [57].

## Materials and Methods

### Materials

[γ-^32^P]ATP was from Perkin-Elmer. All restriction enzymes were from New England Biolabs. All other chemicals were from sources previously described [30], [31], [58].

### Protein purification and *in vitro* interactions of purified proteins

Rhodopsin was purified from cow eyes, phosphorylated, and regenerated by 11-*cis*-retinal generously supplied by Dr. R. K. Crouch (Medical University of South Carolina, Charleston, SC), as described [59]. Bovine arrestins were expressed in E. coli and purified, as described [58], with slight modifications for individual subtypes [8], [10], [14]. Constitutively active MEK1, and inactive ERK2 were expressed in E. coli and purified, as described [60]. ERK2 was activated *in vitro* (phosphorylated at T183 and Y185 by MEK1) as described [60].

### ERK2 interactions with the receptor-bound arrestins

Active (phosphorylated at T183 and Y185 by MEK1) or inactive ERK2 (30 pmol) was preincubated with or without 30 pmol of purified arrestins for 20 min at 30°C, then phosphorylated rhodopsin (50 pmol) was added and incubated in the light (to produce P-Rh*) for 5 min (final volume 0.1 ml). Rhodopsin-containing membranes were pelleted through 0.2 M sucrose cushion, as described [61]. The pellets were dissolved in SDS sample buffer. ERK2 in the pellet (1/300 of each sample) was quantified by Western blot using anti-ERK antibodies (Cell Signaling). Known amounts of purified ERK2 were run on the same gel to generate calibration curve.

### ERK2 interactions with the free arrestins

CNBr-activated Sepharose beads (30 µl) containing 9 µg of covalently attached active phosphorylated (without or with 1 mM ATP) or inactive ERK2 were incubated with 3 µg of indicated purified arrestins in 60 µl of binding buffer (50 mM Tris-HCl, pH 7.4, 100 mM KCl, 1 mM EGTA, 1 mM DTT) for 20 min. at 30°C. The beads were washed twice with 1 ml of ice-cold binding buffer supplemented with 0.01 mg/ml BSA. Bound arrestins were eluted with SDS sample buffer and quantified by Western blot with rabbit polyclonal pan-arrestin antibody, as described [38], [62]. Known amounts of respective purified arrestins were run on each gel to generate calibration curves, as described [63].

### ERK2 phosphorylation by purified MEK1

ERK2 (12 pmol) was incubated with MEK1 (2 pmol) in 0.1 ml of 50 mM Hepes-Na, pH 7.2, 100 mM NaCl, and 0.1 mM [γ-^32^P]ATP in the absence (control) or presence of 4.4 pmol of arrestin-2, arrestin-3, or arrestin-3-(1–393) for 30 min at 30°C. The reaction was stopped by MeOH-precipitation of the proteins. The pellet was dissolved in SDS sample buffer and subjected to SDS-PAGE. The gels were stained, dried, and exposed to X-ray film to visualize radiolabeled bands. ERK2 bands were cut out and ^32^P incorporation was quantified by scintillation counting.

### Co-immunoprecipitation and Western blotting

Monkey kidney COS-7 cells were transfected with the indicated plasmids using Lipofectamine™ 2000 (Invitrogen; Carlsbad, CA), according to the manufacturers protocol (3 µl of Lipofectamine™ 2000 per 1 µg of DNA). 24 hours post-transfection, cells were serum-starved and lysed with lysis buffer (50 mM Tris, 2 mM EDTA, 250 mM NaCl, 10% glycerol, 0.5% Nonidet P-40, 1 mM NaVO_3_, 10 mM N-ethylmaleimide, benzamidine and phenylmethylsulfonylfluoride) on ice for 20 min. Cell debris were pelleted by centrifugation for 10 min at 10,000× g. Lysates were precleared with 30 µl of protein G agarose, followed by incubation with rabbit anti-FLAG antibody for 2 hours and by the addition of 30 µl of protein G agarose beads for 2 hours. The beads were then washed 3 times with lysis buffer, and bound proteins were eluted with Laemmli SDS buffer. In experiments involving ERK2, prior to lysis the cells were treated with 1 mM cross-linking reagent dithiobis(succinimidyl propionate) (DSP; Pierce) for 30 min followed by 2 mM Tris-HCl, pH 7.5, for 15 min at room temperature. The proteins were separated by SDS PAGE (10%) and transferred to polyvinylidene difluoride membrane (Millipore, Bedford, MA). Blots were incubated with primary antibodies from Cell Signaling (mouse anti-HA (6E2) mAb #2367, 1∶1500; mouse anti-p44/42 ERK1/2 (L34F12) mAb #4696, 1∶1000; and mouse anti-p44/42 phospho-ERK1/2 (T202/Y204), (E10) mAb #9106S, 1∶1000), or Sigma (mouse anti-FLAG M2, #F3165, 1∶1500; rabbit anti-FLAG #F7425), followed by anti-mouse horseradish peroxidase-conjugated secondary antibodies from Jackson ImmunoResearch. Protein bands were visualized by enhanced chemiluminescence (ECL, Pierce) followed by exposure to X-ray film. The bands were quantified using VersaDoc with QuantityOne software (Bio-Rad Laboratories).

### Arrestin-dependent ERK activation in cells

#### COS-7 cells

COS-7 cells were transfected using Lipofectamine™ 2000 (Invitrogen; Carlsbad, CA), according to manufacturer's protocol (3 µl of Lipofectamine™ 2000 per 1 µg of DNA) with Flag-tagged arrestin-2 together with ERK2-HA. 24–48 hours post-transfection, cells were serum starved for 24 hours and then treated for 10 min at 37°C with saturating concentrations of isoproterenol (10 µM), epinephrine (10 µM), propranolol (10 µM), alprenolol (1 µM), ICI118551 (1 µM) or carazolol (100 nM). COS-7 were then harvested and lysed in 50 mM Tris, 2 mM EDTA, 100 mM NaCl, 1% Nonidet P-40, supplemented with protease and phosphatase inhibitors cocktails (Roche 04693124001 and 04906845001, respectively) on ice for 20 min.

#### Mouse embryonic fibroblasts (MEFs)

For retrovirus production, human embryonic kidney (HEK) 293T cells were transfected using Lipofectamine™ 2000 (Invitrogen; Carlsbad, CA), according to the manufacturer's protocol (3 µl of Lipofectamine™ 2000 per 1 µg of DNA) with the following constructs: pVPack-GP (Stratagene, 217566), pVack-VSV-G (Stratagene, 217567), together with pFB-arrestin-2, pFB-arrestin-2-3A, pFB-arrestin-2-Δ7, or pFB-GFP (control). 24–48 hours post-transfection, media containing the virus produced by HEK293T cells was collected and used to infect arrestin-2/3 double knockout MEFs (a generous gift of Dr. R. J. Lefkowitz, Duke University) [45]. Fresh virus-containing media was used daily for 3 days. Then MEFs were serum starved for 2 hours and treated with 1 µM ICI118551, a biased ligand of β2AR, which is an inverse agonist of G protein signaling and an agonist of arrestin recruitment [43], or 10 µM β2AR agonist isoproterenol for 10 min at 37°C. MEFs were harvested and lysed in 50 mM Tris, 2 mM EDTA, 100 mM NaCl, 1% Nonidet P-40, supplemented with protease and phosphatase inhibitors cocktails (Roche 04693124001 and 04906845001, respectively) on ice for 20 min.

### Statistical analysis

The data were analyzed using one-way or two-way ANOVA (SAS Institute), as appropriate for particular experimental design, followed by Bonferroni-Dunn post-hoc test with correction for multiple comparisons.
